# Factors associated with the occurrence of injuries requiring hospital transfer among older and working-age pedestrians in Kurume, Japan

**DOI:** 10.1186/s12889-017-4456-8

**Published:** 2017-06-02

**Authors:** Takashi Nagata, Takeru Abe, Ayako Takamori, Yoshinari Kimura, Akihito Hagihara

**Affiliations:** 1 Division of Disaster and Emergency Medicine, Department of Advanced Medical Initiatives, Kyushu University, Faculty of Medical Sciences, 3-1-1, Maidashi, Higashi-ku, Fukuoka-city, 812-8582 Japan; 2 Advanced Critical Care and Emergency Center, Yokohama City University Medical Center, Yokohama, Japan; 3 Biostatistics Center, Kurume University, Kurume, Japan; 4 Graduate School of Literature and Human Science, Osaka City University, Osaka, Osaka Japan; 5 Department of Health Services Management and Policy, Kyushu University Graduate School of Medicine, Fukuoka, Japan

**Keywords:** Pedestrian, Geographical / spatial analysis, Older people, Socioeconomic status

## Abstract

**Background:**

Pedestrian injuries among older people tend to occur near their residence. However, knowledge regarding whether distance travelled from home to the injury site or road environmental/socioeconomic factors affect injury severity remains limited.

**Methods:**

A cross-sectional study was performed using injury registry data from the Kurume City Fire Department, Japan. Distance travelled from home was determined with geographic information system (GIS) software. Data were analyzed for potential association with injury occurrence and severity, with stratification by age. Signal detection analysis using 10 variables was applied to identify factors associated with the occurrence of severe pedestrian injuries.

**Results:**

Among the 545 adult pedestrian injuries reviewed, the factors associated with the occurrence of severe pedestrian injuries for older people and working-age people were evaluated, focusing on the effect of the network distance travelled from home to injury site. Network distance travelled from home to injury site was not associated with the occurrence of severe pedestrian injuries among older people. By applying signal detection analysis, for older people, higher socioeconomic status, wider road width per lane, and higher aging rate in the residential area were significant factors, and for working-age pedestrians, longer network distance travelled between injury place and their residential area and a higher aging rate in the residential area were significantly associated.

**Conclusions:**

To reduce severe pedestrian injuries among older people, improvement of road infrastructure in areas with wider roads, higher socioeconomic status and higher aging rates is required.

## Background

Pedestrian injuries among older people are regarded as a complicated issue for public health and road safety in industrialized countries that are faced with aging societies [1, 2]. Because walking is the principal method of mobility and is also promoted for health among older people, a safe walking environment is important in modern societies [3, 4]. As unprotected road users, pedestrian injuries, especially among older people, tend to be severe because of vulnerability [5, 6]. Additionally, because of fragility, traffic injuries involving older people are associated with longer hospital stays, more time in intensive care units, and poorer outcomes than those for working-age people [7–9]. Pedestrian injuries are highly influenced by the speed of the motor vehicle involved in the crash, and thus controlling the road environment by limiting vehicle speeds is an essential preventive measure [10, 11]. In addition, sociodemographic factors are associated with the occurrence of severe pedestrian injuries. One factor contributing to the occurrence of pedestrian injuries is the distance travelled from home to injury site, which is a proxy measure of risk exposure to traffic in relation to physical activity and behavioral pattern [12–14]. However, evidence examining the distance travelled from home to injury site and injury severity is inconclusive. The National Police Agency, Japan, showed that about 70% of fatal injuries among older pedestrians occur within 500 m from their home [15]. Since this study was based on unknown methods applied by the police, the effects of other factors upon injury outcome are unknown. Further, there are differences in the method of collecting traffic injury data between police, Emergency Medical System (EMS), and hospitals [16, 17]. Thus far, no studies have investigated the association bewteen traveled distance from home to injury site and age of injured people by using EMS records in Japan. Therefore, further studies focusing on the association between the distance from home and occurrence of pedestrian injuries among older people are needed in Japan. Anderson et al. reported that severe injuries more likely occur farther from home, rationalizing the possibility of low-speed collisions on residential streets close to home [18]. Several studies regarding access from injury location to trauma centers in the United States showed that the majority of injuries occur “close to home” and concluded that there were tendencies that the probability of injury occurring “close to home” varied according to specific situations [19, 20]. These findings create difficulty in understanding the factors that contribute to the severity of traffic injury, thereby hampering public health initiatives that could reduce their impact. Thus, studies are needed to identify, more scientifically, the factors associated with severe pedestrian injuries among older people and working-age people.

This study was conducted to reveal how the combination of multiple factors, such as distance travelled from home to injury site and road environment and socioeconomic factors, mutually interact to increase or decrease the possibility of severe pedestrian injuries among older people compared with working-age people. These findings will promote a better understanding of the factors associated with the occurrence of severe pedestrian injuries, followed by development and institution of effective preventive measures.

## Methods

### Study variables, outcomes, and predictors

A cross-sectional study was performed using data from the Kurume City Fire Department (KCFD). Kurume City (population ~ 300,000) is located in Kyushu in southwestern Japan. The economic status of the population in this area is within the average range for Japan, and Kurume-city is a typical suburban setting [21]. KCFD serves Kurume-city and the surrounding cities/towns with both fire and EMS. Data were collected retrospectively from the registry of KCFD from January 1, 2002 to December 31, 2008 regarding traffic injuries resulting in transfer by ambulance to Saint Mary’s Hospital. Saint Mary’s Hospital has 900 beds. The hospital serves as a referral hospital; moderate to severely injured patients are often transferred there from local community hospitals. The population in the catchment area of this hospital is estimated to be about 860,000. Other hospitals in the area also receive injured patients. Saint Mary’s Hospital is a Level 2 facility handling injuries of minor to moderate severity; life-threatening injuries are usually transferred to a Level 1 university hospital in Kurume-city. Annual patient volume in the emergency department (ED) was about 60,000 during the study period, and more than 50% of the total emergency cases were transferred in from outside Kurume City. Injury-related ED visits comprised about 1/6 of the total annual patient load, and traffic-related visits were about 1/5 of all injury visits occurring in Kurume-city and surrounding communities [21]. Over the seven-year study period, 10,633 cases were extracted from the KCFD database. After applying additional criteria based on location (residence was located inside Kurume-city, site of injury was inside Kurume-city, and the network distance travelled could be calculated using geocodes based on the sites of residence and injury), 6607 cases were reviewed. Younger pedestrians, aged 17 years and under, were excluded because previous findings suggested that their behavior and pattern of daily mobility may differ from older people or working-age people [22, 23].

The following characteristics were collected for each injury case: age, gender, injury severity, different types of road user (pedestrians, bicyclists, motorcyclists, or occupants of four-wheel motor vehicles), details of injury location (densely inhabited district, width of road, national road or not, aging rate, area’s socioeconomic status), and location of the residence of the injured person.

Injury severity (minor, severe) was based on triage and diagnosis by the emergency department physician. A minor injury was defined to have occurred when the patient returned home after evaluation and a severe injury was defined as one necessitating admission to the hospital with possible surgical treatment. Cases in which patients were transferred to the hospital but died during transfer or after arrival were also included and categorized as severe injuries in this study. Accident victims who died at the scene were not included in the study because they were not transferred to the hospital by ambulance. The outcome variable assessed in this study was the occurrence of a severe injury.

A densely inhabited district (DID), according to the Japan Statistics Bureau and Statistics Centre, is defined as an area with more than 4000 residents per square kilometer. This statistic is used as an indicator of urbanization. Road width per lane was categorized as follows: less than 3 m, between 3 and 5.5 m, between 5.5 and 13 m, and more than 13 m. In Japan, road width per lane of 3 m is the minimum for ordinary motor vehicles, and national roads have a width over 13 m. Roads were defined as national or not, with national roads being managed by the national government, generally wider and more often used for heavy transport. Injury location was documented as the geographical site to which EMS were dispatched. EMS staff confirmed the location of the injured person’s residence when the patient was transferred to the hospital.

The aging rate was defined as the percentage of people aged 65 years and over, and the mean aging rate was 23% in 2012. In Japan, annual income per household was not available for the study period (2002–2008) from census data. Therefore, area level socioeconomic status was presented as the proportion of the people in the census enumeration district with a household earning an annual income less than 30,000 U.S. Dollars. Annual income less than 30,000 U.S. Dollars is typically considered as lower economic status in Japan [24]. These characteristics were extracted based on the location of the residence of the injured person.

“Network distance travelled” was determined using geographic information system (GIS). This distance-measuring technology enables researchers to gather detailed evidence on road use patterns to identify characteristics that place road users at risk. GIS uses topographical databases and geographical positioning to produce more precise distance measurements [25, 26]. Injury location was derived from the EMS record, and location of the residence of the injured person was derived from the hospital medical record. The distance travelled was calculated using Euclidean distance and network distance travelled. The Euclidean distance (d) was calculated from latitude and longitude on a projected map as d^2^ = (x_1_-x_2_)^2^ + (y_1_-y_2_)^2^. Network distance travelled was defined as the shortest route to a location along a network of transportation routes, by finding the closest point to a given point, and it was calculated by a GIS-based spatial analysis covering the realistic road connectivity conditions and travel speeds. The subjects’ residential locations and geographical sites of injury were geocoded. Thus, network analysis determined the shortest path between the geocoded place of residence and the site of injury on road networks. In this study, the network distance travelled was considered as the distance travelled by the four different modes of transport because, in daily life, people tend to use the shortest path. The network distance travelled therefore is considered as potential estimate-measure to represent how near an injury occurred to the injured person’s home. ArcGIS 9.2 software from Environmental Systems Research Institute (Redlands, CA, USA) was used.

### Statistical analysis

Most previous studies of traffic injuries identified the associated factors using chi-square test with stratification or multivariate regression analysis. These analyses are effective when a model includes the interaction of two variables. However, models addressing the interaction of three or more variables face the issue of multicollinearity, and the higher-order interaction is complex. To resolve these issues, signal detection analysis (SDA) has been proposed by Kraemer [27]. SDA recursively reveals the strongest interaction of factors among groups by using the largest chi-square test statistic and significant probability (*p* < 0.05), and when that factor is absent, the remaining factors are analyzed recursively and repeatedly. SDA is used as a prediction tool such as a recursive partitioning analysis or decision tree analysis, wherein a combination of factors can explain the probability of the outcome [28, 29]. Since road environmental and socioeconomic factors have been identified as mutually associated with the occurrence of pedestrian injuries [30–32], SDA was considered to be optimal to explore the relationship in this study. Since these 10 variables produce thousands of possible combinations, multivariate logistic regression analysis with an interaction model cannot adequately deal with every possible combination.

Patients transported for traffic injuries were divided into two age groups in the analysis, ages 18–64 years (termed working-age people), and over 65 years (termed older people). In Japan, 65 years old is considered as the age of retirement. The World Health Organization and other agencies also define older individuals as people aged 65 years old and over. First, descriptive analyses using the pedestrian injuries extracted from the database were conducted, and t-test and/or tests of independence were used to compare older people and working-age people. SDA (ROC 5.0 software) was used to develop a prediction model for the occurrence of severe pedestrian injuries for older people and working-age people. Based on previous studies and the availability of the data, analysis included ten independent variables, such as basic information (age, gender, time of injury, season), network distance travelled, road environmental factors (national road or not, road width per lane, degree of urbanization at the injury place), and socioeconomic factors (aging rate and proportion of people in a lower socioeconomic status in the census enumeration district.

SDA was performed on the pedestrian injury data to identify factors related to the occurrence of severe injuries compared with minor injuries for older people and working-age people. The SDA partitioning process identifies unknown combinations of certain independent variables to maximize the sensitivity and specificity in predicting the model for severe pedestrian injury. The optimally efficient variable or cut-off point is determined by the maximum weighted-kappa coefficient. After selecting the first variable, the program repeats partitioning for each subgroup using all of the independent variables until the stopping-rules are applied. The stopping-rules for the partitioning processes were triggered when (1) less than 10 patients appeared in a subgroup, (2) no variable was found at a significance level, *p* < 0.05, and (3) less-than-zero values were in the lower limits of the 95% confidence interval of the maximum weighted-kappa coefficient.

Using data for working-age people and older people, the subgroups were compared according to the study variables listed in Table 1. Analysis of variance procedures for continuous variables and tests of independence for dichotomous or discrete variables were used. Two-tailed *p*-values <0.05 indicated statistical significance. Analyses were conducted using R.

**Table 1:** Descriptive statistics of older people and working population

		Older people	Working-age people	*p* value
Age		65 -	18–64	
		(*n* = 229)	(*n* = 316)	
Male		76 (33.2%)	160 (50.5%)	<0.0001
Severely injured		75 (32.8%)	31 (9.8%)	<0.0001
Time	0:00–4:00	3 (1.3%)	28 (8.8%)	<0.0001
	4:00–8:00	33 (14.4%)	26 (8.2%)	
	8:00–12:00	60 (26.2%)	44 (13.9%)	
	12:00–16:00	34 (14.8%)	29 (9.1%)	
	16:00–20:00	78 (34.1%)	112 (35.3%)	
	20:00–24:00	21 (9.2%)	78 (24.6%)	
Season	Spring	71 (31.0%)	86 (27.1%)	0.17
	Summer	54 (23.6%)	70 (22.1%)	
	Fall	37 (16.2%)	76 (24.0%)	
	Winter	67 (29.3%)	85 (26.8%)	
Network distance travelled (mean [SD]) (m)		1128 (1837)	1939 (2487)	<0.0001
National road		60 (18.9%)	43 (18.8%)	0.97
Road width	< 3 m	9 (3.5%)	16 (5.0%)	0.001
	3 < < 5 m	75 (32.8%)	127 (40.1%)	
	5 < < 13 m	107 (46.7%)	96 (30.3%)	
	> 13 m	38 (16.6%)	78 (24.6%)	
Densely inhabited district (mean [SD]) (%)		170 (74.2%)	256 (80.8%)	0.07
Aging rate in the residential area (mean [SD]) (%)		19.64 (5.17)	17.80 (4.93)	0.001
Proportion of lower socioeconomic status in the census enumeration district (mean [SD]) (%)		40.80 (6.50)	41.51 (7.53)	0.25

This study was approved by the Institutional Review Board of Saint Mary’s Hospital, Kurume, Japan.

## Results

Of 6607 cases in the initial database review, 23% were injured as pedestrians. Of these, minors (<18 years old) and non-transferred injury cases including fatalities at the scene were excluded. Thus, 545 pedestrian injuries (including seven pedestrians who died in transit or at the hospital) were selected for further analysis. Descriptive characteristics of the pedestrian injuries are shown in Table 1. Overall, 12.6% were categorized as severely injured (32.8% among older people, and 9.8% among working-age people). Compared with working-age people, the network distance travelled was significantly shorter for older people. Time of injury, road width per lane at the injury location, and aging rate in the residential place were also significantly different between older people and working-age people.

**Fig. 1:**
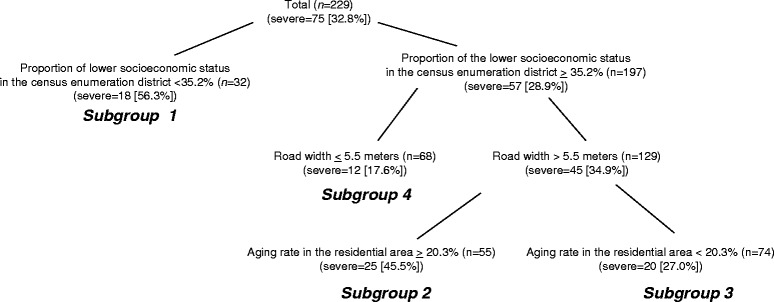
Factors associated with severe pedestrian injuries, ages 65 and over (*n* = 229)

**Fig. 2:**
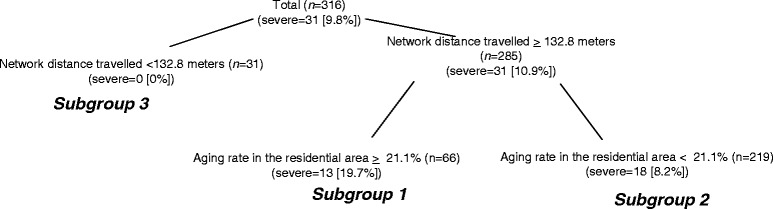
Factors associated with severe pedestrian injuries, ages 18 to 64 (*n* = 316)

SDA identified four distinct subgroups of pedestrian injuries among older people (Fig. 1), and three subgroups among working-age people (Fig. 2). While descriptive names were given to each subgroup, the combination of background information was applied for interpretation of the groups. For example, Subgroup 2 in Fig. 1 is referred to as “lower socioeconomic status, injured in a wide road, and aging rate more than 20.3%.” The percentage of severe injuries within each of the subgroups of older people ranged from 56.3% to 17.6%. Subgroup 1, characterized as having a higher socioeconomic status, had the highest percentage of severe injuries among older people (56.3%). Subgroups 2 and 3 were characterized as having lower socioeconomic status and being injured on wider roads; together they had a combined percentage of severe injuries of 34.9%. These two subgroups were distinguished by another variable, aging rate in the residential area. If the aging rate in the residential area was more than 20.3% (Subgroup 2), the percentage of severe injury was 45.5%, and if less than 20.3% (Subgroup 3), the percentage of severe injury was 27.0%. Subgroup 4 (17.6%, severe injury) was characterized as having lower socioeconomic status and injured on narrow roads.

Among working-age people, three distinct subgroups of pedestrian injuries were identified by SDA, numbered as shown in Fig. 2. The percentage of severe injuries within each subgroup ranged from 19.7% to 0.0%. Subgroups 1 and 2 were characterized as having a network distance travelled longer than 133 m. These subgroups were distinguished by a second variable, aging rate in the residential area. If the aging rate in the residential area was more than 21.1% (Subgroup 1), the percentage of severe injury was 19.7%, and if less than 21.1% (Subgroup 2), the percentage of severe injury was 8.2%. Subgroup 3, characterized as having a shorter network distance travelled, had the lowest percentage of severe injuries among working-age people (0.0%).

Tables 2 and 3 summarize, respectively, the four subgroups of pedestrian injuries among older people and three subgroups among working-age people. In addition to the characteristics revealed by SDA, other details regarding the injury location such as densely inhabited district and national road showed significant variance among groups.

**Table 2:** Descriptive statistics and cross-validation of pedestrian injuries among older people (*n* = 229)

		Subgroup 1	Subgroup 2	Subgroup 3	Subgroup 4	*p* value
		(Proportion of lower socioeconomic status in the residential area < 35.21%)	(Proportion of lower socioeconomic status in the residential area > =35.21%) & (Road width < 5 m)	(Proportion of lower socioeconomic status in the residential area > =35.21%) & (Road width > 5 m) & (Aging rate in the residential area > 20.3%)	(Proportion of lower socioeconomic status in the residential place > = 35.21%) & (Road width > 5 m) & (Aging rate in the residential area < 20.3%)	
Proportion severely injured (%)		32.8	17.6	45.5	27.0	
		(*n* = 32)	(*n* = 68)	(*n* = 55)	(*n* = 74)	
Age (mean)		74.69	74.90	75.98	74.51	0.6443
Male (%)		34.4	30.9	32.7	35.1	0.9572
Time	0:00–4:00 (%)	3.1	1.5	0.0	1.4	0.2489
	4:00–8:00 (%)	12.5	20.6	12.7	10.8	
	8:00–12:00 (%)	12.5	33.8	27.3	24.3	
	12:00–16:00 (%)	15.6	7.4	23.6	14.9	
	16:00–20:00 (%)	40.6	30.9	30.9	36.5	
	20:00–24:00 (%)	15.6	5.9	5.5	12.2	
Season	Spring (%)	15.6	30.9	45.5	27.0	0.1017
	Summer (%)	31.3	20.6	21.8	24.3	
	Fall (%)	28.1	19.1	7.3	14.9	
	Winter (%)	25.0	29.4	25.5	33.8	
Network distance travelled (mean) (unit:m)		1103	796	1690	1028	0.0545
National road (%)		9.4	0.0	29.1	32.4	<0.0001
Road width more than 5 m (%)		50.0	0.0	100.0	100.0	<0.0001
Densely inhabited district (%)		18.8	80.9	78.2	89.2	<0.0001
Aging rate in the residential area (%)		24.2	18.3	23.0	16.4	<0.0001
Proportion of lower socioeconomic status in the census enumeration district (mean [SD]) (%)		32.8	42.1	41.1	42.9	<0.0001

**Table 3:** Descriptive statistics and cross-validation of pedestrian injuries among the working population (*n* = 316)

		Subgroup 1	Subgroup 2	Subgroup 3	*p* value
		(Network distance travelled > = 132.84) & (Aging rate in the residential place > = 21.05)	(Network distance travelled > = 132.84) & (Aging rate in the residential place <21.05)	(Network distance travelled <132.84)	
Proportion severely injured (%)		19.7	8.2	0.0	
		(*n* = 66)	(*n* = 219)	(*n* = 31)	
Age (mean)		43.24	44.22	44.42	0.8739
Male (%)		47.0	51.8	48.4	0.7644
Time	0:00–4:00 (%)	9.1	9.1	6.5	0.9906
	4:00–8:00 (%)	9.1	8.2	6.5	
	8:00–12:00 (%)	16.7	13.2	12.9	
	12:00–16:00 (%)	7.6	9.5	9.7	
	16:00–20:00 (%)	37.9	35.0	32.3	
	20:00–24:00 (%)	19.7	25.0	32.3	
Season	Spring (%)	28.8	27.3	22.6	0.6710
	Summer (%)	24.2	21.8	19.4	
	Fall (%)	27.3	23.6	19.4	
	Winter (%)	19.7	27.3	38.7	
Network distance travelled (mean) (unit:m)		3032	1877	53	<0.0001
National road (%)		28.8	17.7	6.5	0.0231
Road width more than 5 m (%)		60.6	54.1	48.4	0.8552
Densely inhabited district (%)		71.2	84.1	77.4	0.0589
Aging rate in the residential area (%)		23.9	16.0	17.3	<0.0001
Proportion of lower socioeconomic status in the census enumeration district (mean [SD]) (%)		39.8	41.8	39.8	0.0767

## Discussion

By applying SDA to 545 adult pedestrian injuries, we investigated the factors associated with the occurrence of severe pedestrian injuries for older people and working-age people, focusing on the effect of the network distance travelled from home to injury site. The most significant finding is that the network distance travelled from home to injury site is not associated with the occurrence of severe pedestrian injuries among older people, while it is associated for working-age people. For working-age people, the measured network distance travelled from home to injury site was 133 m. A possible explanation for this result is that most severe pedestrian injuries among working-age people did not occur near their home. This hypothesis requires further research.

The second significant factor was socioeconomic status for the occurrence of severe injuries among older people. The percentage of severe injuries in the high and low socioeconomic status areas showed distinct difference, 56.3% and 27.0%, respectively. Lower socioeconomic status has typically been considered as a factor contributing to the occurrence of severe traffic injuries. Lower socioeconomic status is sometimes associated with reduced ability to purchase private vehicles, or less organized infrastructure such as traffic signals and road blocks, which may contribute to the occurrence of pedestrian injuries in a lower socioeconomic status area. This study found the opposite. One potential explanation is that older people with higher socioeconomic status are more socially independent and thus more mobile [30, 32], and more exposed to traffic and involved in traffic incidents when walking outside their home. The third significant factor was road width per lane. Severe pedestrian injuries occurred more frequently on roads more than 5.5 m wide. In the wider roads, since the speed of vehicles was faster and the impact of a collision greater, this may be associated with more severe pedestrian injuries among older people [11]. It also tends to take a longer time for older people to cross wider roads, and older pedestrians might be more exposed to road traffic. The fourth significant factor was the aging rate in the residential area. It is possible that areas with many older people contain a larger proportion of individuals who have difficulty traveling because of weakened physical capabilities associated with aging.

The network distance travelled from home to injury site was the most significant factor for severe pedestrian injuries among working-age people. The second significant factor was the aging rate in the residential area. The cut-off percentage for the aging rate was 21.1. This could be interpreted as working-age people tend to be severely injured at the area with higher aging population. This mechanism can not be appropriately explained and further studies are needed.

These results suggest that preventive measures for older pedestrian injuries in Japan should be focused on areas with higher socioeconomic status and aging rates in addition to roads with wider lane widths. These factors are characteristics of the community; therefore, road users and community members should be aware of the high-risk situation for pedestrian injuries. Recognition of road width as a risk factor for severe pedestrian injuries among older people can generate attention within the community for targeted traffic safety promotion [31]. Further, in communities with a higher aging rate, wider roads, or higher socioeconomic status, improvement of road infrastructure such as road design, signals, and road blocks in wider roads will be effective to reduce the risk of pedestrian injuries among older people [32].

This study includes several limitations. First, data validity should be taken into account. The data were collected from a single institution, and did not represent population-based incidence of pedestrian injury in the area. The interpretation of the study results should be done with care. Additionally, the possibility of selection bias should be considered because there are several hospitals with emergency departments in the area, and all the traffic injuries that occurred in Kurume and the surrounding areas were not captured. Since Japan is the society aging most rapidly in the world, the findings regarding traffic safety among older people in Japan may have implications for other areas. However, since these findings are based on data from one emergency hospital in a middle-sized city in Japan, the external validity of the findings is limited. Second, the proportion of the people in an area with households earning an annual income less than 30,000 U.S. Dollars was used to determine socioeconomic status. Although annual income less than 30,000 U.S. Dollars is considered as lower economic status in Japan, the proportion of the people in the census enumeration district with a household earning an annual income less than 30,000 U.S. Dollars is not widely used in public health research, and the interpretation should be made with caution. However, it is difficult to collect individual socioeconomic status in research in Japan, and estimation was needed. Third, a limited number of variables was used in SDA. Adding other variables such as clinical information, weather, and detailed injury situation might provide different study results. Fourth, the data were generated between 2002 and 2008, and the social conditions and demography at that time, particularly the aging rate and percentage of low-income households, may not reflect more recent trends. Fifth, fatalities at the scene were not included in the analysis, and risk factors associated with pedestrian fatalities or not surviving a collision could not be identified in this study.

The study also has several strengths. First, this is one of the first studies to analyze the effect of the network distance travelled between injury place and residential place for pedestrians. Second, SDA could resolve the issue of multicolinearity and interaction of the variables in the multivariate regression analysis. Selected variables were purely associated with the occurrence of severe pedestrian injuries.

## Conclusions

For older people, higher socioeconomic status, wider road width per lane, and higher aging rate in the residential area are significantly associated with severe injuries, and network distance travelled between the place of injury and the residential area was not a significant contributor to injury. Reduction of severe pedestrian injuries among older people could be achieved by improving infrastructures of wider roads in the areas with a higher aging population, including higher socioeconomic areas where older pedestrian mobility might be greater.

## Abbreviations

DID: Densely inhabited district
EMS: Emergency medical service
GIS: Geographic information system
KCFD: Kurume city fire department
SDA: Signal detection analysis
